# Approach to Abnormal Liver Biochemistries in the Primary Care Setting

**DOI:** 10.7759/cureus.56541

**Published:** 2024-03-20

**Authors:** Rajesh R, Aditya Sangameshwar, Yi Yuan Tan, Kevin Teh Kim Jun, Tat Yean Tham, Mark Cheah Chang Chuen

**Affiliations:** 1 Gastroenterology and Hepatology, Singapore General Hospital, Singapore, SGP; 2 Family Medicine, National University Hospital, Singapore, SGP; 3 Family Medicine, Frontier Healthcare, Singapore, SGP

**Keywords:** liver biochemistry, gastroenterology, liver, approach to lft, primary care, liver function test, hepatology

## Abstract

Liver biochemistries are commonly ordered in the primary care setting, and they may return abnormal even in an asymptomatic patient. Primary care physicians need to have a systematic way of interpreting any derangement in these tests so that further investigations, referrals, and management can be arranged appropriately. This review dwells into patterns of liver biochemistry derangement, common aetiologies to consider, history and examinations that are required, initial investigations to order, and when to refer urgently to the emergency department.

## Introduction and background

In the primary care setting, liver biochemistries are routinely ordered for various groups of patients, and a wide range of abnormalities can be detected even in otherwise healthy patients. Abnormal liver biochemistry is often the first clue of liver diseases in an asymptomatic patient [1]. The constituents of a standard liver panel can vary depending on the laboratory [2]. The most commonly ordered liver biochemistries include alanine aminotransferase (ALT), aspartate aminotransferase (AST), alkaline phosphatase (ALP), and bilirubin [3].

There is a rise in the prevalence of liver diseases globally, particularly driven by the spectrum of steatotic liver diseases (SLDs), particularly metabolic dysfunction-associated steatotic liver disease (MASLD) [4]. Hence, primary care physicians need to have a systematic way of interpreting any derangement in these tests so that further investigations, referrals, and management can be arranged appropriately [5].

## Review

Physiology of liver biochemistries

ALT and AST are enzymes involved in the transfer of amino groups alanine and aspartate to ketoglutaric acid, respectively [6]. They are both present in hepatocytes and are released into the bloodstream upon hepatocyte injury. However, they differ in their distribution in the body. ALT is predominantly found in the liver and is considered liver-specific. Meanwhile, AST is additionally found in the cardiac muscle, skeletal and smooth muscle, kidneys, and brain. AST elevation can be expected in rhabdomyolysis or myocardial infarction [7]. While the reference range for normal ALT can vary from laboratory to laboratory, there has been interest globally in revising the cutoff to a lower value to increase the sensitivity of the test. It is hypothesized that previous studies that established ALT cutoffs could have included patients with subclinical liver disease; hence, currently, the American Association for Study of Liver Diseases (AASLD) recommends a reference range of 35 IU/L for males and 25 IU/L for females [8].

ALP is a zinc metalloproteinase that catalyzes the hydrolysis of phosphate esters [9]. It is distributed in the canalicular membrane of hepatocytes, bone, kidney, intestine, and placenta. Amongst these extrahepatic sites, the bone is the most common source. ALP elevation of hepatic origin is accompanied by a concomitant rise in gamma-glutamyl transferase (GGT), another canalicular enzyme. Alternatively, fractionating ALP into heat-labile (bone source) and heat-stable components can be a useful guide [10]. 

Bilirubin is a byproduct of the breakdown of heme [11], which can exist in unconjugated or conjugated forms. Unconjugated bilirubin is brought to the liver bound to albumin where it undergoes conjugation by uridine 5’-diphosopho-glucoronosyl-transferase (UDP-GT) and and hence becomes water-soluble for excretion. Elevation in unconjugated bilirubin can reflect hemolysis or a failure of conjugation, whereas elevation in conjugated bilirubin is often due to biliary obstruction or parenchymal liver pathology.

Causes of abnormal liver biochemistries

Abnormal liver biochemistries can be broadly categorized into a few patterns, namely, hepatocellular, cholestatic, mixed, and isolated raised bilirubin. The R factor is a useful tool initially developed for drug-induced liver injury but subsequently recommended as an objective way to define the patterns of injury. It is calculated as (ALT/upper limit of normal (ULN) reference of ALT) / (ALP/ULN reference of ALP) [12]. While the R factor is a useful tool, it is but a guide as it is a static tool. Any available previous results should also be sought out and analyzed as the trend may provide insight into the temporal evolution of the disease and can help clinch the diagnosis. A complete history with emphasis on medications [13], occupation, and travel history is equally important to understand any abnormal liver tests. Table 1 summarizes the main patterns and the differentials to consider for each pattern.

**Table 1:** Patterns of liver injury and differentials

Injury type	R value	Derangements	Differentials	
Hepatocellular	>5	Predominantly raised ALT and AST	Parenchymal causes	1. Metabolic dysfunction associated steatotic liver disease(MASLD), 2. alcohol-associated liver disease, 3. viral hepatitis, 4. autoimmune hepatitis, 5. drug-induced liver injury, 6. hereditary causes
			Vascular causes	1. Ischemic, 2. congestive, 3. Budd-Chiari, 4. sinusoidal obstruction syndrome (SOS)
Cholestatic	<2	Predominantly raised ALP and GGT	Biliary obstruction	1. Choledocholithiasis, 2. malignancy: cholangiocarcinoma, pancreatic head cancer, 3. primary and secondary sclerosing cholangitis
			Biliary epithelial damage	1. Hepatitis, 2. cirrhosis
			Intrahepatic cholestasis	1. Drug-induced liver injury, 2. total parenteral nutrition, 3. sepsis, 4. primary biliary cholangitis
Mixed	>2 and <5		All of the above	All of the above
Isolated raised bilirubin	NA	Raised conjugated or unconjugated bilirubin	Unconjugated bilirubin	1. Gilbert syndrome, 2. hemolysis
			Conjugated bilirubin	1. Consider cholestatic injury causes, 2. Dubin-Johnson/Rotor syndromes

Workup of abnormal liver biochemistries

Hepatocellular Pattern

**Figure 1:**
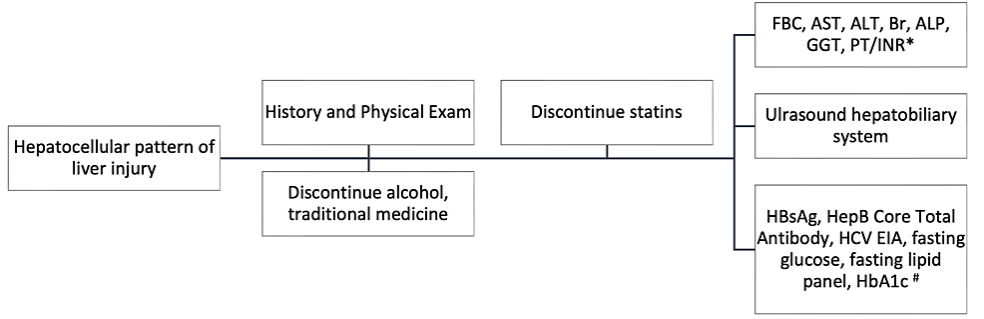
Initial approach to the hepatocellular pattern of liver biochemistry * FBC: full blood count, AST: aspartate transaminase, ALT: alanine transaminase, Br: bilirubin, ALP: alkaline phosphatase, GGT: gamma-glutamyl transpeptidase, PT: prothrombin time, INR: international normalized ratio # HBsAg: hepatitis B surface antigen, HCV EIA: hepatitis C enzyme immunoassay, HbA1c: hemoglobin A1c

In a hepatocellular pattern of liver injury, the degree of aminotransferase elevation can be further subclassified as mild (2-5x ULN), moderate (5-15x ULN), and severe (>15x ULN) [14]. It is more likely that mild to moderate injuries are encountered in primary care, whereas severe injuries typically >1000 IU/L are referred to a tertiary center. Figure 1 details our approach.

When a hepatocellular pattern of liver injury is encountered, a systematic review of the patient’s history of alcohol consumption (baseline drinking habit, drivers of the drinking habit, and whether there has been bingeing behavior) [15], metabolic risk factors, body mass index, and medications including alternative medications in the last three months should be asked for. A history of viral hepatitis should be sought. Given that a majority of chronic hepatitis B is acquired via mother-to-child transmission [16], a family history of hepatitis B is pertinent. A history of prior hepatitis B status can be sought around key life events, for example, for females during pregnancy or as a part of pre-employment. Risk factors for hepatitis C, including tattoos, prior transfusions, and intravenous drug abuse, need to be screened [17].

A complete physical examination looking for features of chronic liver diseases, such as the presence of jaundice, ascites, gynecomastia, and Dupuytren's contracture, is suggested. Common initial investigations include screening for hepatitis B serology, hepatitis C serology, full blood count, lipid panel, HbA1c, and an ultrasound of the hepatobiliary system [18]. Common medications such as statins can be continued as long as the AST/ALT levels are not above five times the ULN under close monitoring [19]. When there is doubt about whether a medication is contributory, resources are available online, such as LiverTox, to verify the likelihood and usual patterns of injury [20]. The De Ritis ratio (AST:ALT) can be useful where a ratio of 2 or more is consistent with alcoholic liver disease [21]. 

The presence of hepatitis B surface antigen (HBsAg) for more than six months is indicative of chronic hepatitis B infection. Surveillance for HCC with six monthly US HBS and serum alpha-fetoprotein is recommended in hepatitis B patients with cirrhosis, Asian men over 40 years of age, Asian women over 50 years of age, patients with first-degree family members with hepatocellular carcinoma, patients with hepatitis D virus, and African Americans. The decision for treatment of hepatitis B takes into account the presence of advanced fibrosis or cirrhosis, ALT level, and HBV DNA load [22]. From the primary care perspective, hepatitis B patients with persistent elevation of ALT or features of cirrhosis on ultrasound should be referred to a hepatologist for further evaluation and treatment.

For suspected chronic hepatitis C, the first line of testing should be HCV antibody. If this test returns positive, it could indicate either an active or past infection. An HCV RNA test should then be sent off to confirm whether the former is likely [23]. When there is an active infection, a referral to a hepatologist should be made for treatment. Meanwhile, in suspected acute hepatitis C, initial antibody testing may be negative due to latency in antibody formation; hence, the RNA should be sent directly if the clinical suspicion is high [24].

Non-alcoholic/metabolic fatty liver disease is typically picked up coincidentally on abdominal imaging, such as ultrasound. The findings of a hyperechoic, “echo-bright” liver are suggestive of hepatic steatosis [25]. A history of alcohol should be sought, and other causes of liver injury should be excluded. If not known, the patient should be evaluated for metabolic syndrome, with an assessment for hypertension, diabetes, and dyslipidemia [26]. Anthropomorphic measures, such as weight, BMI, and waist circumference, should be performed. In primary care, non-invasive scores, such as the FIB-4 (comprising age, ALT, AST, and platelet count) or NAFLD score, are helpful clinical tools to guide management. Such scores allow clinicians to determine if the patient is at a low, high, or indeterminate risk of advanced fibrosis [27]. Hepatic fibrosis in patients with fatty liver disease is the key determinant for adverse liver-related outcomes. Therefore, patients who are at a high or indeterminate risk of advanced fibrosis warrant a hepatology referral for further assessment of fibrosis, which typically involves transient elastography in the form of a Fibroscan®, shearwave elastography, or magnetic resonance elastography [28]. 

Cholestatic Pattern

**Figure 2:**
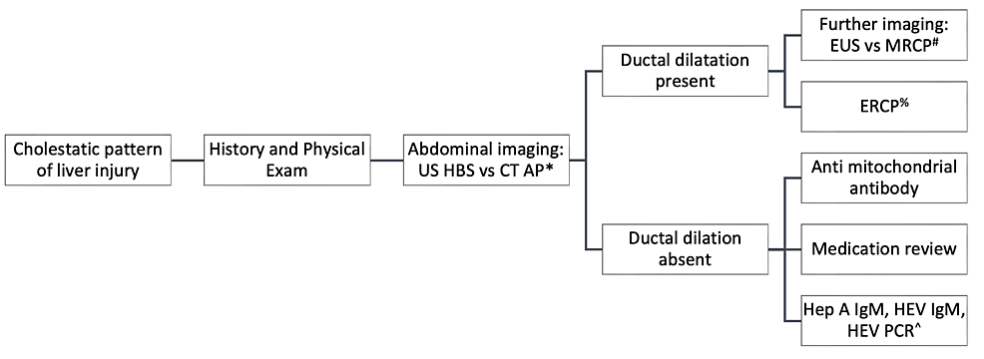
Initial approach to the cholestatic pattern of liver biochemistry *US HBS: ultrasound hepatobiliary system, CTAP: computed tomography of the abdomen and pelvis. # EUS: endoscopic ultrasound, MRCP: magnetic resonance cholangiopancreatography. % ERCP: endoscopic retrograde cholangiopancreatography. ^ Hep A IgM: hepatitis A IgM, HEV IgM: hepatitis E IgM, HEV PCR: hepatitis E PCR

A cholestatic pattern of liver injury usually results in a predominantly raised ALP and GGT with or without raised bilirubin. Etiological causes can be broadly divided into causes of bile duct obstruction, biliary epithelial damage, or functional impairment in the bile flow [29]. Our approach to the cholestatic pattern of liver injury is presented in Figure 2.

History needs to probe for symptoms of biliary colic, which may suggest choleliathiasis, the presence of itch that may represent bile acid malabsorption seen in chronic cholestasis, red flags for pancreatobiliary malignancy such as painless jaundice with weight loss, and new-onset diabetes in a non-obese elderly patient. A history of exposure to medications including antibiotics (commonly augmentin) [30,31] and over-the-counter and alternate medications [32] should be sought. Physical exams would be targeted to look for scleral icterus, xanthelasma, and evidence of symptomatic pruritus, such as scratch marks and palpable abdominal masses. 

The most important investigation would be abdominal imaging, which can start with an ultrasound of the hepatobiliary system (US HBS) [33]. In a patient who has a history and examination worrisome for malignancy, it would be prudent to refer to a restructured hospital for consideration of computed tomography of the abdomen and pelvis (CTAP) upfront. 

Although autoimmune liver disease is a consideration especially in females with a family history of other autoimmune diseases, given the costs of autoimmune testing, these are best evaluated by a hepatologist where appropriate. Regardless, a presentation of itch in a female with xanthelasma and hepatomegaly should raise suspicion for primary biliary cholangitis (PBC) [34,35].

Mixed and Isolated Hyperbilirubinemia

A mixed pattern of liver injury is a broad entity for which both typical causes of hepatocellular and cholestatic injuries need to be considered. Further investigations will be guided by the history and examination as detailed above.

When isolated hyperbilirubinemia is picked up, it needs to be further characterized as indirect (unconjugated) or direct (conjugated). Hemolysis and Gilbert syndrome [36] are the most common causes of indirect hyperbilirubinemia. A history of intermittent episodes of jaundice that is present during periods of illness followed by resolution is typical for Gilbert syndrome [37]. In this case, no further testing is necessary. Meanwhile, for hemolysis, we would need to send off further workup with haptoglobin, lactate dehydrogenase, reticulocyte count, and blood film among other further investigations [38].

Indication for urgent referrals

Majority of the liver biochemistry derangements can be worked up non-urgently in the outpatient setting, but some have to be referred to the emergency department of a restructured hospital urgently. This would include the following situations: 1) acute liver failure: new onset liver injury (<28 weeks) with PT >15 s and hepatic encephalopathy [39,40]; 2) elevation of ALT and AST >10x ULN; 3) ascending cholangitis for consideration of biliary decompression; 4) obstructive jaundice with constitutional symptom.

## Conclusions

Abnormal liver biochemistries are commonly encountered in the primary care setting. A thorough history and physical examination are important to elucidate the underlying aetiology, and further investigations are required. Categorizing based on the R factor can give a structured approach to the initial workup of deranged liver biochemistry, which can be initiated in the primary care setting before onward referral to a specialist. If there is suspicion of severe acute liver injury, acute liver failure, cholangitis, and obstructive jaundice, a timely referral to the nearest emergency department would be important.
